# Perspectives on applying immuno-autonomics to rheumatoid arthritis: results from an online rheumatologist survey

**DOI:** 10.1007/s00296-022-05122-3

**Published:** 2022-04-21

**Authors:** Dimitrios A. Pappas, Christine Brittle, Andrew Concoff, Andrew J. Holman, Dennis Takasugi, Joel M. Kremer

**Affiliations:** 1 CorEvitas LLC, Waltham, MA USA; 2 Corrona Research Foundation, Waltham, MA USA; 3 HealthiVibe, a Division of CorEvitas LLC, Arlington, VA USA; 4 Inmedix Inc., Normandy Park, WA USA; 5 Pacific Rheumatology Associates, Inc. PS, Seattle, WA USA; 6 United Rheumatology, Hauppauge, NY USA

**Keywords:** Arthritis, Rheumatoid, Rheumatologists, Surveys and questionnaires, Autonomic nervous system, Immuno-autonomics, Heart rate variability

## Abstract

The term “immuno-autonomics” has been coined to describe an emerging field evaluating the interaction between stress, autonomic nervous system (ANS), and inflammation. The field remains largely unknown among practicing rheumatologists. Our objective was to evaluate the perspectives of rheumatologists regarding the role of stress in the activity and management of rheumatoid arthritis (RA). A 31-item survey was conducted with 231 rheumatologists. Rheumatologists were asked to assess the role of stress in rheumatoid arthritis (RA) disease activity and were provided with information regarding immuno-autonomics. They were asked to consider how immuno-autonomics resonated with their patient management needs. The majority of rheumatologists are eager to better understand non-response, believe that stress biology and ANS dysfunction interfere with disease activity, and embrace the theory that measurement of ANS via next-generation HRV may be able to evaluate autonomic dysfunction and the biology of stress. Rheumatologists are open to the idea that quantitative measurement of ANS function using next-generation HRV can be a helpful tool to RA practice. The majority agree that ANS state influences RA disease control and that quantitative measures of ANS state are helpful to RA practice. Rheumatologists also agree that patients with poor ANS function may be at risk for not responding adequately to conventional, biologic, or targeted synthetic DMARDs. Almost all would use an in-office test to quantitatively measure ANS using next-generation HRV. This study shows that rheumatologists are open to embracing evaluation of ANS function as a possible tool in the management and treatment of RA.

**Supplementary Information:**

The online version contains supplementary material available at 10.1007/s00296-022-05122-3.

## Introduction

Rheumatoid arthritis (RA) is an autoimmune disease that affects 1–2% of the US population [1]. Ground-breaking therapies (biologics and targeted synthetic drugs) have demonstrated efficacy to adequately treat the disease in > 50% of patients [2]. However, a significant subset of RA patients do not respond to the approved therapies for RA. Multiple switches between successive drugs that fail to control RA disease activity are not unusual, and contribute to patient and physician frustration in addition to increasing treatment costs. In 2016 dollars, the direct, indirect, and intangible US annual costs of RA were estimated to be $56 billion [3]. Biologic therapy was a large portion of this cost, with failure to respond among RA patients increasing costs even further—costing approximately $6000 additional per patient per year [4].

Research to identify patients who will not respond to therapy has shown variable results. Several biomarkers—genetic or otherwise—have been evaluated; however, in most cases, results have not been validated consistently [5]. In an attempt to better understand non-response, new strategies have been under focus. The term “immuno-autonomics” has been coined to describe an emerging field evaluating the role of ANS status in RA disease activity. Immuno-autonomics evaluates the interaction between stress, autonomic nervous system (ANS) activity, and inflammation [6]. Although it has received some attention, the field remains largely unknown among practicing rheumatologists.

Accumulating evidence supports a linkage between ANS function, stress, and increased disease activity. An overactive sympathetic nervous system seems to be related to stress and enhanced inflammation. In contrast, the parasympathetic nervous system may result in better disease control [7–10]. Next-generation heart rate variability (HRV) evaluation has been proposed as a measure of ANS state [11] and has been applied in rheumatology. (Next-generation HRV is an expansion of HRV that includes measurements of sympathetic/ parasympathetic activity and a tension index [12, 13]). Next-generation HRV has been associated with inflammatory cytokine levels and flares of systemic lupus erythematosus [14], and has been correlated with RA activity [15]. Briefly, lower HRV indicates a predominant sympathetic activity and is associated with more inflammation, while higher HRV is associated with predominant parasympathetic activity and less inflammation.

In this research, we sought to understand the familiarity of practicing rheumatologists with immuno-autonomics as a field relevant to the treatment of RA patients and to evaluate their interest to better understand how ANS may be associated with rheumatoid arthritis disease state.

## Methods

Data were collected via a cross-sectional survey of US rheumatologists. A 31-item survey was designed by HealthiVibe, a division of CorEvitas, LLC, a research and consulting company which specializes in surveys for patients and healthcare professionals. Several rheumatologists with an interest in immuno-autonomics served as expert reviewers for the survey design. The survey was fielded from August 31 to October 1, 2021, using the online survey platform Alchemer. The survey instrument was cognitively pretested with 12 practicing rheumatologists using a think-aloud technique prior to being finalized to ensure content and construct validity. The complete survey instrument is available electronically as supplemental information to this article (Supplement 1). The study was reviewed by the Sterling Institutional Review Board, and a letter of exemption as non-human subjects research was received. All respondents were asked to review an informed consent statement prior to participating. Respondents were advised that participation was voluntary and that they could withdraw at any time. All questions were required, and participants could only go forward in the survey. IP address controls were used to ensure that no participant completed the survey more than once.

### Respondents

Rheumatologists were recruited from a panel (M3 Global Research) and a rheumatology care management organization (United Rheumatology), and were offered a small gratuity ($20–$40) for completing the survey. All survey contacts were made electronically via email message. Survey panel members were invited to participate directly via M3; rheumatologists recruited via the care management organization received a message stating, “United Rheumatology is helping to field a survey to learn more from rheumatologists about their treatment of rheumatoid arthritis (RA) patients. Please help us by taking this short 10-min survey by clicking the link below.” At the beginning of the survey, respondents were told that the purpose of the survey was to seek “feedback from physicians about their treatment of rheumatoid arthritis patients.” Study qualification requirements included: a primary medical specialty of rheumatology; primarily treat adult patients; been in clinical practice three or more years; and evaluate at least 15 RA patients per month.

### Survey methodology

**Fig. 1:**
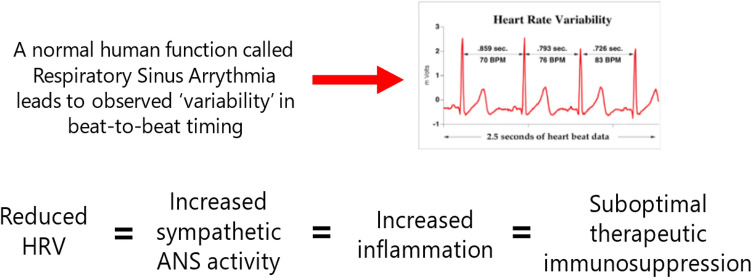
Model shown to survey respondents depicting connection between reduced HRV and suboptimal therapeutic immunosuppression

Questions were organized into four main sections. The first collected demographic information and current practice management for RA patients. This included demographic and practice setting-related questions. It also included questions about how the interviewee’s RA patients currently respond to treatment and rheumatologists’ interest in new treatments for RA patients. The second section provided information on the potential role of stress in RA, including information on stress biology and findings from a patient survey indicating a large percentage of patients believe stress can trigger their RA. Survey respondents rated their level of agreement with four statements about stress and RA, and answered questions about how they currently assess and address stress in their practice. The third section provided information on the possible role of evaluation of the ANS for the management of RA patients, including the use of next-generation HRV as a tool to assess autonomic state. Pertinent references (about 40) were provided, as well as several paragraphs of information reviewing the available literature. Rheumatologists also were shown a model (see Fig. 1) and a narrative description of several studies showing an association between reduced HRV (indicative of increased sympathetic ANS activity), which in turn may be associated with increased inflammation and suboptimal therapy results. Next-generation HRV was presented to interviewees as an accepted tool to measure autonomic state. Survey respondents rated their level of agreement with statements about ANS and RA, and their interest in being able to quantitatively measure ANS using next-generation HRV. The final section asked respondents to consider their interest in a test to measure autonomic state using next-generation HRV and to indicate on which groups of RA patients they would consider using such a test. All materials shown to respondents are available in Supplement 1.

### Statistical analysis

Survey data were reviewed to check for response errors or concerning response patterns (e.g., users who consistently selected the top answer category to each question, or users who completed in an extremely short amount of time). No invalid responses were noted. Only complete survey responses were used in the analysis.

Descriptive statistics were used to characterize trends in the data. Key survey variables were evaluated in a cross-tabulation analysis against demographic variables including years in practice, gender, race, practice characteristics, and practice setting. Chi-square analyses were used to identify statistically significant differences across respondent groups (e.g., male vs. female respondents). No weighting of survey data was performed.

## Results

### Participant demographics

**Fig. 2:**
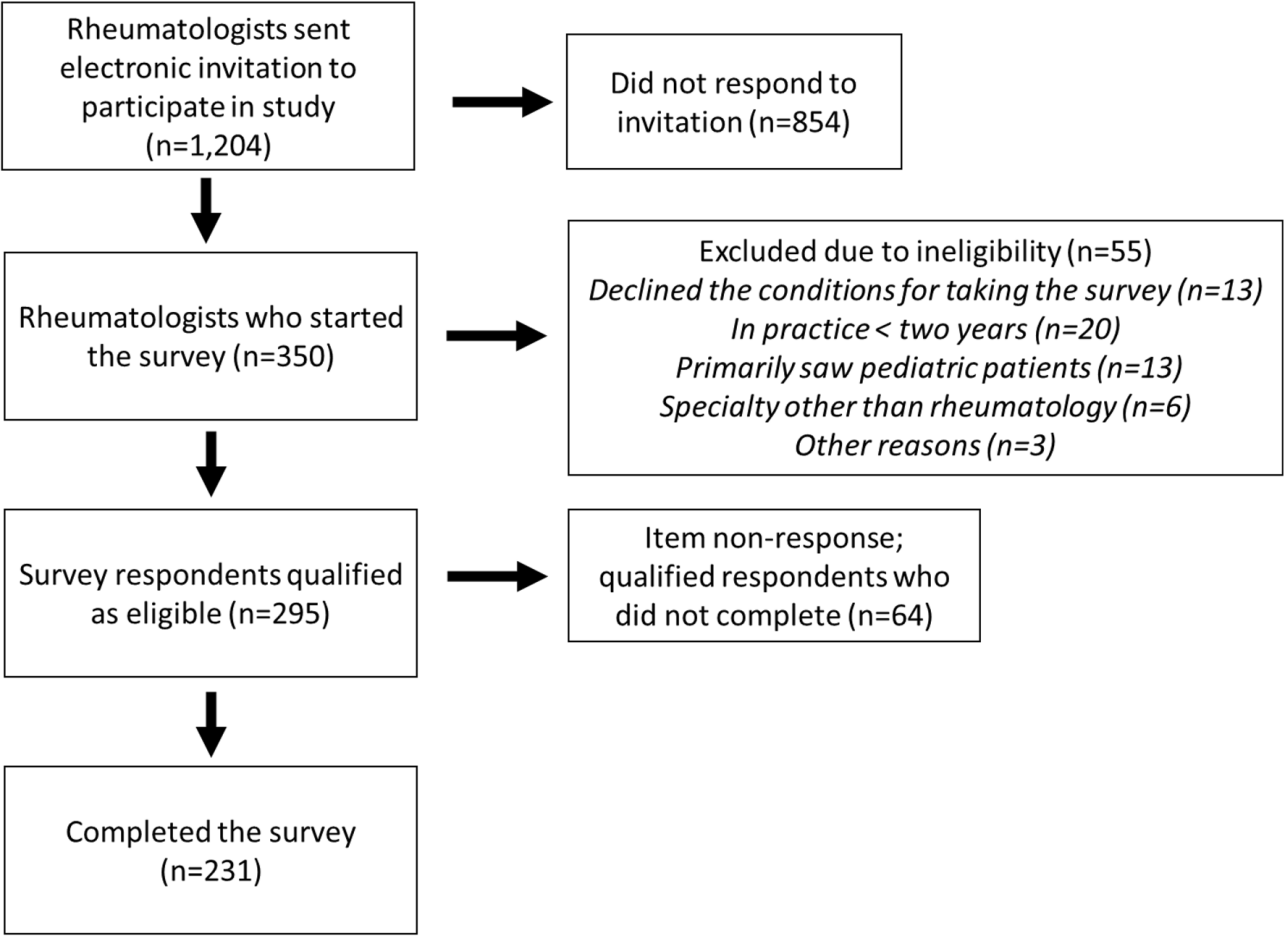
Survey response flow chart

The survey was sent to 1204 potential respondents (see Fig. 2). A total of 350 respondents began the survey (participation rate = 29%); 55 were excluded due to ineligibility, resulting in an eligibility rate of 84% (295). (Among those who were ineligible: *n* = 13 declined the conditions for taking the survey; *n* = 20 had been in practice less than 2 years; *n* = 13 primarily saw pediatric patients; *n* = 6 had a specialty other than rheumatology; and *n* = 3 were screened out for other reasons.) Among eligible respondents, the majority (231/295; 78%) completed the survey. On average, the survey took 11 min to complete.

As shown in Table 1, respondents are primarily male (66%) and white (60%). Most (63%) have been in practice 11 or more years, and more than half (60%) see 81 or more adult RA patients per month. About a fifth (17%) practice in an academic setting. These results are similar to the demographics reported in the 2015 ACR Workforce Study [16], which found that 59.2% of rheumatologists are male, and 20% practice in an academic setting. Rheumatologists are actively engaged in continuing education with 74% saying they participate in multiple CME activities per year and 89% subscribing to rheumatology journals.

**Table 1:** Demographics and practice characteristics of survey respondents

		**%**	*n*
Gender	Male	66%	153
	Female	29%	66
	Other or prefer not to answer	5%	12
Race or ethnicity	White	60%	139
	Black or African-American	2%	5
	Hispanic or Latino	2%	4
	Asian	22%	50
	Other or prefer not to answer	15%	35
Years in practice (since fellowship)	3–5 years	15%	34
	6–10 years	22%	52
	11–20 years	22%	51
	More than 20 years	41%	94
Adult RA patients per month	15–40	8%	18
	41–80	32%	75
	81–100	28%	63
	101 or more	32%	75
Practice setting	Academic	17%	40
	Nonacademic	83%	191
Practice type	Solo	23%	52
	Single specialty	38%	89
	Multispecialty	39%	90
Geographic location	Rural	6%	13
	Suburban	48%	111
	Urban	46%	107
US region	Midwest	17%	39
	Northeast	35%	82
	Southeast	22%	50
	Southwest	9%	21
	West	17%	39
Self-described early adopter of medical advances	Yes	75%	174
	No	25%	57
Attends conferences or meetings	Multiple times per year	35%	80
	Annually	47%	110
	Less often than annually	18%	41

### Current treatment approaches for RA patients

Rheumatologists were asked to classify their RA patients according to their current disease activity. A mean of 34% (median = 30%) of patients are categorized as having moderate to high disease activity (range from 0 to 86%). Rheumatologists also were asked to classify for what percentage of their RA patients is it difficult to achieve low disease activity or remission. Their answers range from 2 to 95%, with a mean of 28% (median = 25%) (Table 2).

**Table 2:** Rheumatologists’ perceptions of RA treatment and interest in new treatments

		%	*n*
For what percentage of your RA patients is it difficult to achieve low disease activity or remission?	0–10%	12%	28
	11–20%	36%	84
	21–30%	26%	61
	31–50%	17%	39
	51% or more	8%	19
There is a need for new tools or tests to assess why some RA patients don’t respond to conventional, biologic, or targeted synthetic DMARDs	Completely agree	62%	144
	Somewhat agree	27%	62
	Neither agree nor disagree	5%	10
	Somewhat disagree	3%	9
	Completely disagree	3%	6
I would be interested in a new tool or test to identify RA patients who are less likely to respond to conventional, biologic, or targeted synthetic DMARDs	Completely agree	63%	146
	Somewhat agree	27%	63
	Neither agree nor disagree	5%	10
	Somewhat disagree	4%	9
	Completely disagree	1%	3
I would like to better understand disease pathways that may predict treatment outcomes for RA patients	Completely agree	56%	130
	Somewhat agree	36%	82
	Neither agree nor disagree	5%	12
	Somewhat disagree	2%	4
	Completely disagree	1%	3

Rheumatologists express interest in new tools or tests to predict treatment outcomes for RA patients, as well as ways to better understand disease pathways (Table 2). Most respondents (89%) agree there is a need for new tools or tests to assess why some RA patients do not respond to conventional, biologic, or targeted synthetic DMARDs. Likewise, 90% agree they would be interested in a new tool or test to identify RA patients who are less likely to respond to treatment. More than 9 in 10 (92%) also agree they would like to better understand disease pathways that may predict treatment outcomes for RA patients.

### Role of stress in RA treatment

Rheumatologists accept stress as playing an important role in disease activity and treatment for RA patients (see Table 3). Most (84%) agree that increased patient stress is related to higher disease activity, and 87% agree stress can make RA patients less likely to respond to treatment. Almost 9 in 10 (89%) agree they would like to know more about the role of stress in RA treatment, and 85% would like to be able to measure the effects of stress and the biology underlying stress. Most rheumatologists (90%) report that they currently assess patient stress in their RA patients, primarily via patient conversation (87%). However, only about a third (32%) always or often treat patient stress in their RA patients. Common treatment approaches include lifestyle adjustments such as diet or exercise (67%), in-office counseling (50%), and medications (45%).

**Table 3:** Rheumatologists’ beliefs about the role of stress in RA treatment and current approaches to treating stress

		%	*n*
Increased patient stress is related to higher disease activity for RA patients	Completely agree	43%	99
	Somewhat agree	41%	94
	Neither agree nor disagree	14%	33
	Somewhat disagree	2%	5
	Completely disagree	< 1%	0
Increased patient stress can make RA patients less likely to respond to treatment	Completely agree	37%	86
	Somewhat agree	50%	114
	Neither agree nor disagree	9%	22
	Somewhat disagree	4%	9
	Completely disagree	< 1%	0
I would like to know more about the role of stress biology in RA patients	Completely agree	50%	115
	Somewhat agree	39%	91
	Neither agree nor disagree	8%	19
	Somewhat disagree	2%	4
	Completely disagree	1%	2
I would like to be able to easily and accurately measure the effect of stress biology in RA patients	Completely agree	44%	101
	Somewhat agree	41%	95
	Neither agree nor disagree	11%	26
	Somewhat disagree	4%	8
	Completely disagree	< 1%	1
How do you currently assess patient stress (i.e., stress biology) in your RA patients?	Via patient conversation	87%	200
	Via patient-reported outcomes	23%	52
	Via physical examination	28%	64
	Don’t currently assess	10%	23
How often do you currently treat patient stress (stress biology) in your RA patients?	Always	7%	16
	Often	26%	59
	Sometimes	50%	116
	Never/don’t assess stress	17%	40
How do you currently address patient stress (stress biology)?	Lifestyle adjustments	67%	154
	In-office counseling	50%	115
	Medications	45%	103
	Complementary therapies	41%	95
	External counseling	39%	90

A few group differences are noted. Those in an academic setting are more likely to report they use patient-reported outcomes to assess stress (31% vs. 20%, *p* < 0.05) as are those who attend conferences multiple times per year vs. less often (28% vs. 16–23%, *p* < 0.05). Rheumatologists in practice from three to five years are less likely to assess patient stress always or often versus those in practice longer (27% vs. 37–39%, *p* < 0.05). Similarly, stress is assessed more frequently among those who identify themselves as early adopters (39% vs. 28%, *p* < 0.05) or who attend multiple conferences per year (43% vs. 26–35%, *p* < 0.05).

### Role of autonomic nervous system for RA patients

**Fig. 3:**
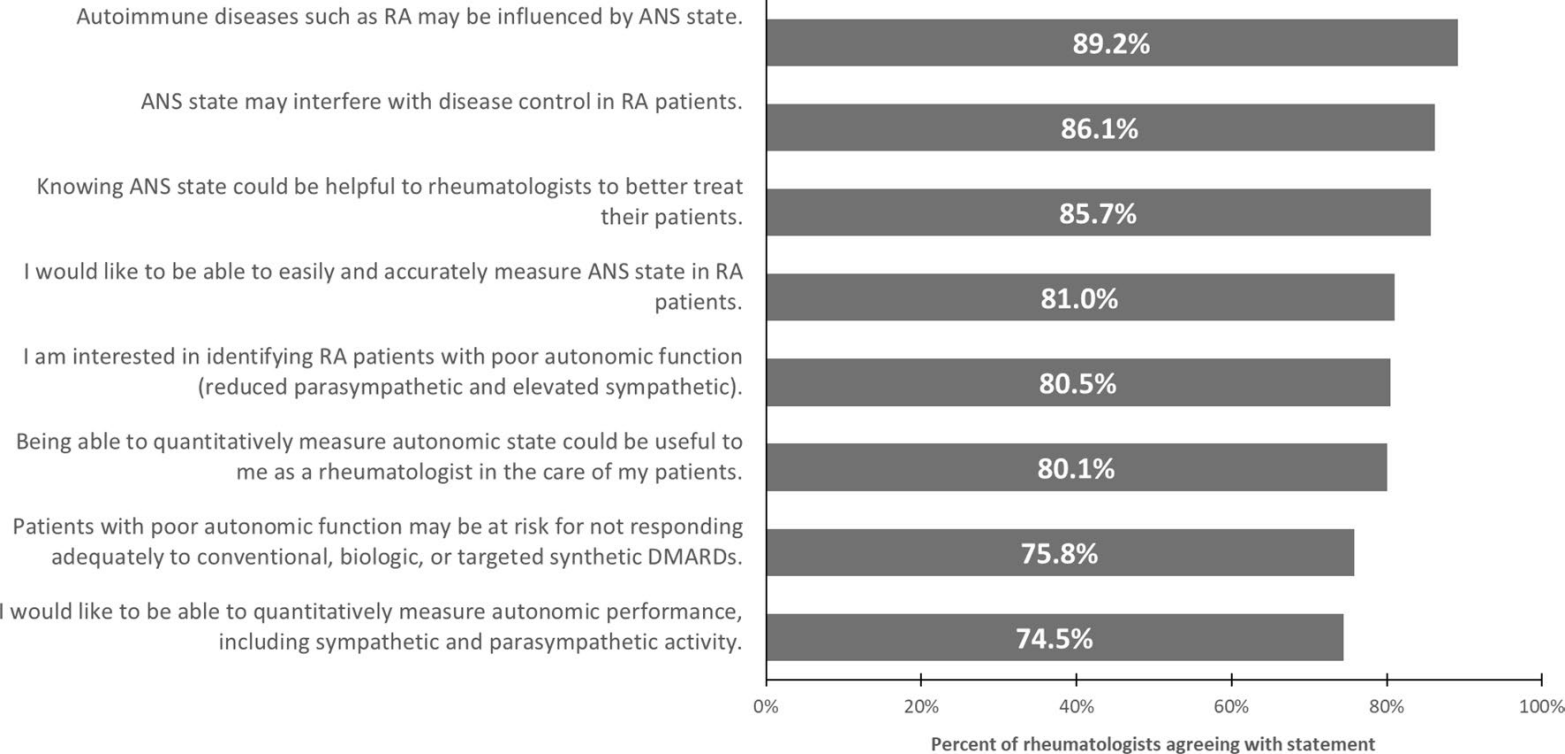
Percentage of rheumatologists agreeing with statements about autonomic nervous system (ANS) state and heart rate variability (HRV)

Rheumatologists were presented with information on the role of the ANS, including five-minute HRV as an established metric of ANS state, which can be measured quantitatively [11]. With the information provided, respondents were asked to assess whether they agree or disagree with various statements about ANS, HRV, and RA (see Fig. 3). There is a strong level of agreement with all statements given, indicating a belief among rheumatologists that ANS state influences RA presentation and treatment. At least 8 in 10 respondents agree with the majority of the statements, including that: “Autoimmune diseases such as RA may be influenced by ANS state” (89%), “ANS state may interfere with disease control in RA patients” (86%), “Knowing ANS state could be helpful to rheumatologists to better treat their patients” (86%), “I would like to be able to easily and accurately measure ANS state in RA patients” (81%), “I am interested in identifying RA patients with poor autonomic function” (81%), and “Being able to quantitatively measure autonomic state (stress biology) could be useful to me as a rheumatologist in the care of my patients” (80%). In addition, about three-quarters agreed that, “Patients with poor autonomic function may be at risk for not responding adequately to conventional, biologic, or targeted synthetic DMARDs” (76%), and “I would like to be able to quantitatively measure autonomic performance, including sympathetic and parasympathetic activity” (75%).

Several group differences are noted, especially among self-described early adopters. Early adopters (89% vs. 80%, *p* < 0.05) and those in practice more than 20 years (91% vs. 81–86%, *p* < 0.05) are more likely to believe ANS state may interfere with disease control. Early adopters also are more likely to think autoimmune diseases are influenced by ANS state (92% vs. 82%, *p* < 0.05). Those in practice 20 or more years are more likely to think knowing ANS state could be helpful to their practice (91% vs. 82–84%, *p* < 0.05). Early adopters are more interested in being able to quantitatively measure autonomic performance (78% vs. 67%, *p* < 0.05) and are more likely to see being able to measure ANS state as useful to their practice (83% vs. 74%, *p* < 0.05). Early adopters (81% vs. 64%, *p* < 0.05) and rheumatologists in a multispecialty practice (82% vs. 71–73%, *p* < 0.05) are more likely to think patients with poor ANS function are at risk of not responding adequately to treatment.

### Interest in a test to measure ANS function

Rheumatologists were asked to consider how interested they would be in an in-office test to measure ANS function. Most rheumatologists are interested in such a test, with 84% saying they are extremely, very, or moderately interested in such a test. Interest is higher among self-identified early adopters and among those who go to multiple conferences per year.

**Fig. 4:**
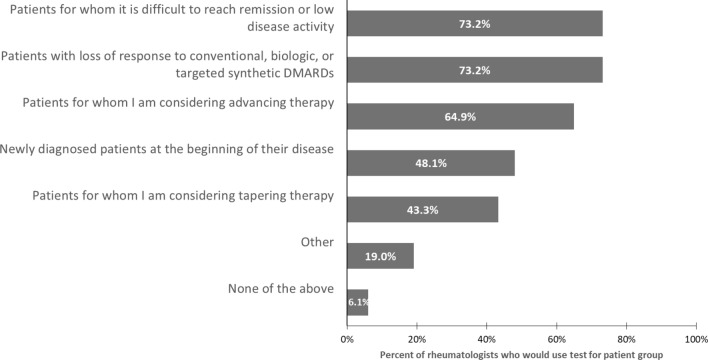
Percentage of rheumatologists who would consider using a test to measure the state of the autonomic nervous system (ANS) for specific groups of patients

Rheumatologists also were asked to consider whether they would use such a test and for which patient groups they would consider using such a test (Fig. 4). Almost all (94%) indicate they would use such a test. Most commonly, rheumatologists say they would use such a test on patients for whom it is difficult to reach remission or low disease activity (73%), as well as for patients with loss of response to conventional, biologic, or targeted synthetic DMARDs (73%). About two-thirds (65%) also would consider such a test on patients for whom they are considering advancing therapy, and about half (48%) would consider using a test on newly diagnosed patients. In addition, 4 in 10 (43%) would use a test on patients for whom they are considering tapering therapy.

## Discussion

Mechanisms explaining why a significant subset of RA patients fail to respond to conventional, biologic, or targeted synthetic DMARDs have not been fully elucidated. Immuno-autonomics is a field investigating the relationship between stress, autonomic nervous function, and disease activity. It proposes that suboptimal autonomic function may interfere with disease control. Conceptually, if autonomic dysfunction is “measured” and “corrected,” disease activity may be easier to ameliorate. Next-generation HRV has been suggested as a method to evaluate autonomic status.

One small, prospective, double-blind study showed that next-generation HRV evaluation at day 0 correlated with 52-week biologic treatment (TNFi) in patients with RA or psoriatic arthritis (PsA) [17]. Patients were segregated into quartiles of ANS function. Patients in ANS dysfunction (lowest parasympathetic activity quartile) had 52-week ACR20/50/70 outcomes of 40%/12%/0%, respectively. On the other hand, patients with favorable ANS profiles (highest parasympathetic activity quartile) had 52-week ACR20/50/70 outcomes of 100%/88%/65%, respectively. For the 52-week ACR70 outcome, the receiver operating characteristic (ROC) area under the curve (AUC) for the parasympathetic measure and a sympathetic measure called tension index were 0.926 and 0.918, respectively [18].

In our survey, we attempted to initially evaluate the perceptions of practicing rheumatologists regarding the role of stress in RA disease activity. After collecting this initial assessment, we provided information regarding immuno-autonomics. Subsequently, we evaluated the extent to which immuno-autonomics as measured using next-generation HRV resonates with physicians’ patient management needs.

The results reinforce the need for better treatment of RA patients. The majority of rheumatologists are eager to better understand non-response, believe that stress biology and ANS dysfunction interfere with disease activity, and embrace the theory that measurement of ANS via next-generation HRV may be able to evaluate autonomic dysfunction and the biology of stress.

Rheumatologists are open to the idea that quantitative measurement of ANS function using next-generation HRV can be a helpful tool to RA practice. The majority agree that ANS state influences RA disease control and that quantitative measures of ANS state are helpful to RA practice. Rheumatologists also agree that patients with poor ANS function may be at risk for not responding adequately to conventional, biologic, or targeted synthetic DMARDs. Almost all would use an in-office test to quantitatively measure ANS using next-generation HRV.

Regarding strengths of this study, the survey was designed, structured, and administered according to best practice survey design principles [19]. In addition, respondents were representative of US practicing rheumatologists on demographic traits such as gender and practice setting. Furthermore, we investigated the association between specific characteristics (e.g., continuing education, self-identification as early adopter, participating in meetings, etc.) and survey responses, including the eagerness to embrace new theories for disease management.

Among the weaknesses of this study, the survey presented a favorable view of immuno-autonomics and the use of next-generation HRV to measure ANS state. The brief literature review presented to respondents did not include possible criticisms against immuno-autonomics. Including such information could have impacted the results. In addition, it is possible that a survey such as this presenting a product that is not associated with any possible adverse events—in our case, a theory that can assist in disease evaluation or control—may be perceived more favorably by respondents.

In summary, immuno-autonomics is a field with which most rheumatologists are not familiar. However, evidence supporting the role of stress biology and ANS function in the treatment of RA deserves consideration. If we accept that stress and ANS dysfunction play a role in RA non-response, then evaluation and management of stress as measured via next-generation HRV could benefit the patient and perhaps facilitate disease control. Of course, more studies are needed to substantiate the theory proposed by immuno-autonomics and the role of next-generation HRV as a tool to evaluate ANS function and perhaps guide disease management. This study shows that rheumatologists are open to embracing ANS function as measured via next-generation HRV as a possible tool in the management and treatment of RA.

## Supplementary Information

Below is the link to the electronic supplementary material.

Supplementary file1 Survey questionnaire completed by rheumatologists (DOCX 1349 KB)

## Data Availability

The data underlying this article will be shared on reasonable request to the corresponding author and following ethical or other needed approval.
