# Supplementary material for: Perspectives on applying immuno-autonomics to rheumatoid arthritis: results from an online rheumatologist survey
Source: Rheumatol Int. 2022 Apr 21;42(9):1555–64. doi: 10.1007/s00296-022-05122-3 (PMC9349152; doi:10.1007/s00296-022-05122-3)
Supplement: Supplementary file 1 — Supplementary file1 Survey questionnaire completed by rheumatologists (DOCX 1349 KB) [file 296_2022_5122_MOESM1_ESM.docx]

# SUPPLEMENTAL MATERIAL 1

## Survey questionnaire completed by rheumatologists

**[Survey/Browser Title]** Rheumatologist Perspectives Survey

**[Note: Question numbers, section headings and bolded programming instructions will not appear onscreen. All questions required and accept only one response, unless otherwise noted.]**

**[INTRODUCTION LANDING PAGE]**

Thank you for your interest in this survey. We are seeking feedback from physicians about their treatment of rheumatoid arthritis patients.

**[INFORMED CONSENT STATEMENT]**

**By entering the survey link you understand and agree to the following:**

- I understand that the aim of this research is to gain my views for market research purposes in the development of marketing campaigns, educational materials, and the development of new commercial products AND IS NOT INTENDED AS A PROMOTIONAL EXERCISE.
- I agree that anything I see or read during this research should be treated as confidential. Any information presented during the course of this research is done solely to explore reactions to such information and should be assumed to represent hypotheses about what can be said about a product or disease area. It should not be used to influence decisions outside the research setting.
- I understand that I can withdraw at any time.
- I understand that any information I disclose will be treated in the strictest confidence and that the results of the research will be aggregated to provide an overall picture of attitudes. My feedback will remain confidential. My personal information will not be passed to any other organization without my permission.

() I accept these conditions for taking the survey.

() I do not accept these conditions for taking the survey. [**TERMINATE**]

**[SCREENING QUESTIONS]**

Thank you. We will begin with a few questions about you and your practice.

1. What is your primary medical specialty? **[ROTATE, BUT KEEP ‘OTHER’ LAST]**

() Rheumatology

() Family practice/primary care **[TERMINATE]**

() Internal medicine **[TERMINATE]**

() Other **[TERMINATE]**

1. How many years have you been in clinical rheumatology practice (time since completing fellowship)?

() 2 or fewer years **[TERMINATE]**

() 3 to 5 years

() 6 to 10 years

() 11 to 20 years

() More than 20 years

1. What percentage of patients in your practice are pediatric (age 17 and under)?

() None (0%)

() 1% to 10%

() 11% to 50%

() 51% or more **[TERMINATE]**

1. How many adult rheumatoid arthritis (RA) patients do you see per month?

() 14 or fewer **[TERMINATE]**

() 15 to 40

() 41 to 80

() 81 to 100

() 101 or more

1. What is your primary practice setting?

() Academic **[CAP AT 20% OF RESPONDENTS]**

() Nonacademic

**DISQUALIFICATION LANGUAGE:**

Thank you for your interest. Unfortunately, you do not qualify for this survey.

**[CURRENT PRACTICE AND CURRENT UNDERSTANDING OF IMMUNO-AUTONOMICS AND HRV]**

Thank you. The following questions are about your current treatment approaches for your rheumatoid arthritis (RA) patients.

1. Please classify your RA patients according to their current disease activity. You can base your categorization on any disease activity metric you use in your practice. For reference, the cut-offs for DAS28 and CDAI are below.

**DAS28:**

Low disease activity (LDA) is defined as a score of 2.6 to 3.2

Remission is defined as a score of <2.6

**CDAI (Clinical Disease Activity Index):**

LDA is defined as a score between 2.8 and 10.0

Remission is defined as a score of <2.8

__ % moderate or high disease activity

__ % low disease activity (but not remission)

__ % remission

**[ACCEPT ONLY NUMERIC RESPONSES BETWEEN 0-100. MUST TOTAL 100.]**

1. In your experience, for what percentage of your RA patients is it difficult to achieve low disease activity or remission? ____% **[ACCEPT ONLY NUMERIC RESPONSE BETWEEN 0-100]**
2. Despite the advancements in rheumatoid arthritis therapeutics, a considerable proportion of patients do not reach low disease activity or remission. How much do you agree or disagree with each of the following statements? **[ROTATE]**

|  | **Completely disagree** | **Somewhat disagree** | **Neither agree nor disagree** | **Somewhat agree** | **Completely agree** |
| --- | --- | --- | --- | --- | --- |
| There is a need for new tools or tests to assess why some RA patients don’t respond to conventional, biologic, or targeted synthetic DMARDs. | | | | | |
| I would be interested in a new tool or test to identify RA patients who are less likely to respond to conventional, biologic, or targeted synthetic DMARDs. | | | | | |
| I would like to better understand disease pathways that may predict treatment outcomes for RA patients. | | | | | |

**For the next several questions, please consider the following information:**

In 2019, the National Society for Rheumatoid Arthritis in the UK (a leading patient advocacy group) surveyed patients with RA. They found that 97% of RA patients believe stress can trigger flares.


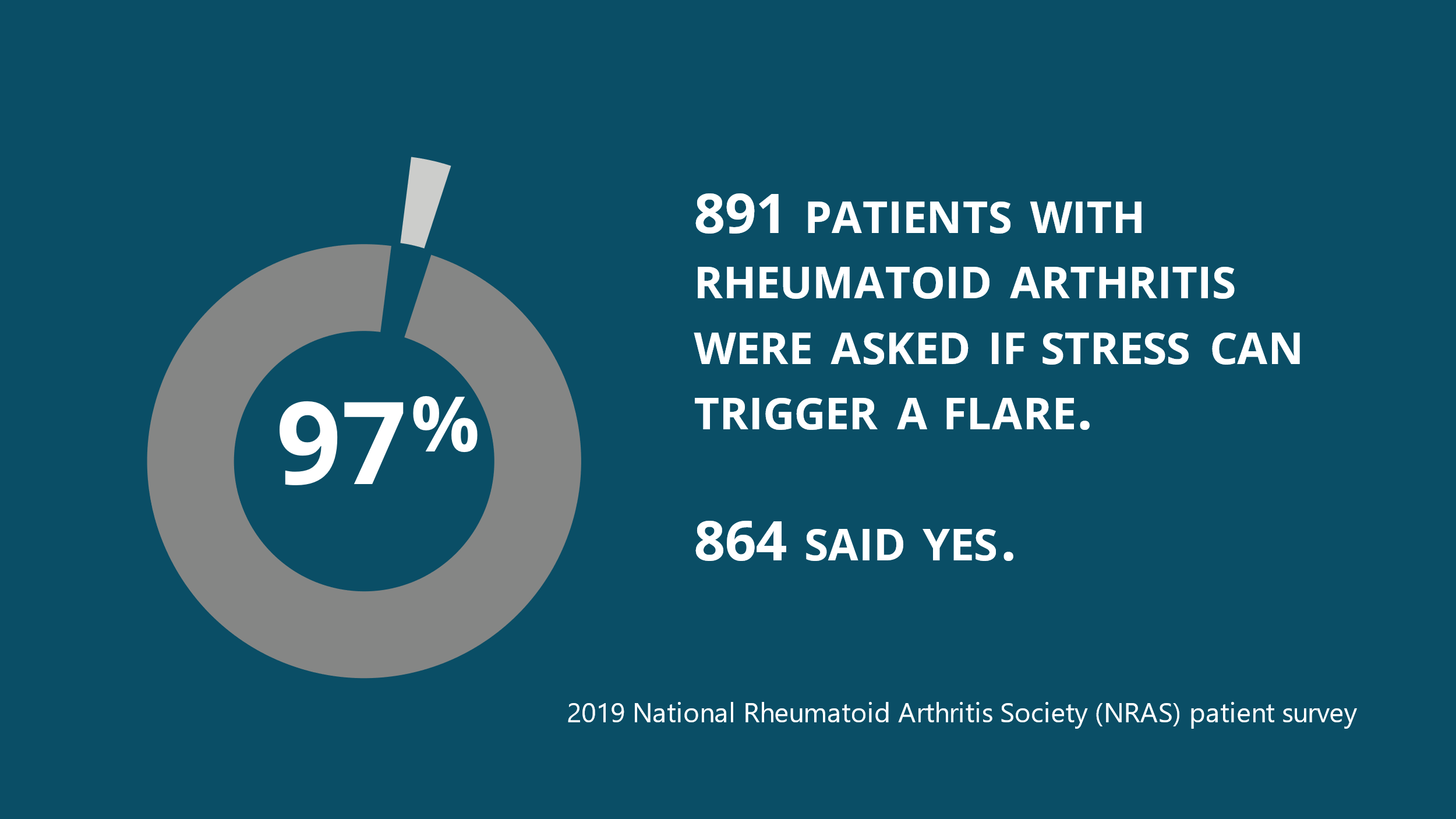


The biology of stress (stress biology) is managed by the endocrine system and autonomic nervous system (ANS). The endocrine (hormonal) system acts in minutes/hours/days, while the ANS acts in milliseconds. The ANS is further organized into sympathetic (fight-or-flight) and parasympathetic (rest-and-restore) components, which constantly compete for dominance, depending on bodily needs.

1. Many patients self-report that stress leads to flares for their rheumatoid arthritis. How much do you agree or disagree with each of the following statements? **[ROTATE]**

|  | **Completely disagree** | **Somewhat disagree** | **Neither agree nor disagree** | **Somewhat agree** | **Completely agree** |
| --- | --- | --- | --- | --- | --- |
| Increased patient stress is related to higher disease activity for RA patients. | | | | | |
| Increased patient stress can make RA patients less likely to respond to treatment. | | | | | |
| I would like to know more about the role of stress biology in RA patients. | | | | | |
| I would like to be able to easily and accurately measure the effect of stress biology in RA patients. | | | | | |

1. How do you currently assess patient stress (i.e., stress biology) in your RA patients? Please select all that apply.

[] Via patient conversation

[] Via patient-reported outcomes (PROs)

[] Via physical examination

[] Via other methods

[] I don’t currently assess stress biology in my RA patients **[IF SELECTED, SKIP TO ANS TEXT BLOCK]**

1. How often do you currently treat patient stress (stress biology) in your RA patients?

() Always

() Often

() Sometimes

() Never **[IF SELECTED, SKIP TO ANS TEXT BLOCK]**

1. How do you currently address patient stress (stress biology)? Please select all that apply.

[] In-office counseling

[] External counseling

[] Medications

[] Lifestyle adjustments (e.g., diet, exercise)

[] Complementary therapies (e.g., massage, acupuncture)

[] Other: Please specify ___________

**[ANS TEXT BLOCK]**

**For the next set of questions, please consider the following information about the role of the autonomic nervous system and rheumatoid arthritis patients:**

The autonomic nervous system or ANS (sympathetic and parasympathetic nervous systems) plays a key role in the overall regulation of the immune system, and stress biology impacts autoimmune disease activity and treatment outcomes.

In 2000, the NIH published a 40-page review with over 450 references highlighting how the autonomic nervous system is connected to immune function, including its impact on pro-inflammatory cytokines, T-cell populations, maturation, and migration to target (Elenkov et al., 2000). Since then, further research has continued to investigate this connection, with numerous studies looking at the function of the ANS in rheumatoid arthritis patients.


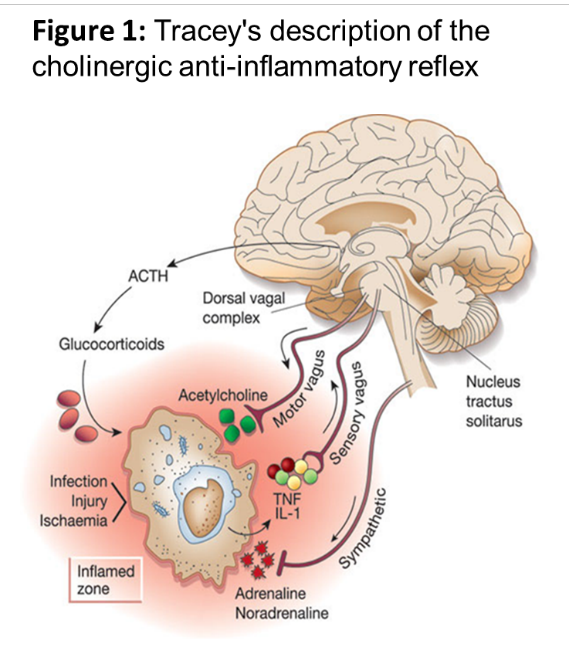


In 2002 (*Nature*), Tracey described the cholinergic anti-inflammatory reflex. The vagus nerve relies on afferent fibers (75%) to monitor inflammation, and efferent fibers (25%) to attenuate inflammation. The vagus nerve monitors systemic inflammation and signals, through α7 nicotinic acetylcholine receptors, to reduce systemic inflammation (see Figure 1).

Since 2002, numerous authors have proposed ways to attenuate inflammatory arthritis activity by reactivating a dormant cholinergic anti-inflammatory reflex. Based on the available literature, relevant findings to date include:

- **Stress is associated with autoimmune disease activity.** A review of articles looking at the effects of stress in inflammatory rheumatoid diseases (de Brouwer et al., 2010) found that “alterations in autonomic function in response to stress are correlated with disease severity.” The authors noted that “real-life stressors could contribute to the maintenance or exacerbation of rheumatic diseases.”
- **Unfavorable autonomic state is common among RA patients. An indicator of ANS dysfunction is low heart rate variability (HRV).** A review article by Adlan et al. (2014) reported that about 60% of rheumatoid arthritis patients have autonomic nervous system dysfunction, characterized by low heart rate variability (HRV), reduced parasympathetic activity, and elevated sympathetic activity. Enhanced sympathetic reactivity among rheumatoid arthritis patients has been reported elsewhere (Parnes et al., 2005; Evrengul et al., 2004).
- **Autonomic state may predict the development and severity of RA.** Koopman et al. (2017) reported that “autonomic dysfunction precedes and predicts arthritis development in subjects at risk of developing seropositive RA.” Ingegnoli et al. (2020) reviewed the link between the autonomic nervous system and rheumatoid arthritis and reported that “disruption of the autonomic system has been linked to RA onset and activity.”
- **The connection between ANS and immune activity has been termed immuno-autonomics.** A growing series of publications has developed this concept from association to causation (see next page).

The following references have developed the concept of immuno-autonomics from association to causation. Please review, then select next to continue the survey.

**References on immuno-autonomics, 2000 to present:**

1. Elenkov IJ, Wilder RL, Chrousos GP, Vizi ES. **The sympathetic nerve – an integrative interface between two supersystems: the brain and the immune system.** Pharmacol Rev 2000;52:595-638.
2. Tracey KJ, Czura CJ, Ivanova S. **Mind over immunity.** FASEB J 2001;15:1575-1576.
3. Tracey KJ. **The inflammatory reflex.** Nature 2002;420:853-859.
4. Pavlov VA, Wang H, Czura CJ, Friedman SG. **The cholinergic anti-inflammatory pathway: a missing link in neuroimmunomodulation.** Mol Med 2003;9(5-8):125134.
5. Eskandari F, Webster JI, Sternberg EM. **Neural immune pathways and their connection to inflammatory diseases.** Arthritis Res Ther 2003;5:251-265.
6. Shimizu M, Tachibana N, Hagasaka Y, Goto M. **Obstructive sleep apnea in RA patients and effect of CPAP on RA activity** [abstract]. Arthritis Rheum 2003;48(suppl):S114.
7. Harle P, Bongartz T, Scholmerich J, Muller-Ladner U, Straub RH. **Predictive and potentially predictive factors in early arthritis: a multidisciplinary approach.** Rheumatology 2005;44:426-433.
8. Czura CJ, Tracey KJ. **Autonomic neural regulation of immunity.** J Intern Med 2005;257:156-166.
9. Pavlov VA, Tracey KJ. **Controlling inflammation: the cholinergic anti-inflammatory pathway.** Biochem Soc Trans 2006;34(pt6):1037-40.
10. Vassilopoulos D, Mantzoukis D. **Dialogue between the brain and the immune system in inflammatory arthritis.** Ann NY Acad Sci 2006;1088:132-138.
11. Thayer JF, Sternberg E. **Beyond heart rate variability: vagal regulation of allostatic systems.** Ann NY Acad Sci 2006;1088:361-372.
12. Sloan RP, McCreath H, Tracey KJ, Sidney S, Liu K, Seeman T. **RR interval variability is inversely related to inflammatory markers: the CARDIA study.** Mol Med 2007;13(3-4):178-184.
13. Ofek K, Krabbe KS, Evron T, Debecco M, Nielsen AR, Brunnsgaad H, Yirmiya R, Soreq H, Pedersen BK. **Cholinergic status modulations in human volunteers under acute inflammation.** J Mol Med 2007;85(11):1239-51.
14. Anichkov DA, Shostak NA, Ivanov DS. **Heart rate variability is related to disease activity and smoking in rheumatoid arthritis patients.** Int J Clin Pract 2007;5:777-783.
15. Mravec B. **Autonomic dysfunction in autoimmune disease: consequence of cause?** Lupus 2007;16:767-768.
16. Holman AJ, Ng E. **Heart rate variability predicts anti-tumor necrosis factor therapy response for inflammatory arthritis.** Auton Neurosci. 2008 Dec 5;143(1-2):58-67. doi: 10.1016/j.autneu.2008.05.005. Epub 2008 Jul 16. PMID: 18632310.
17. Mravec B, Ondicova K, Valaskova Z, Gidron Y, Hulin I. **Neurobiological principles in the etiopathogenesis of disease: when diseases have a head.** Med Sci Monit 2009;15(1): RA6-16.
18. Straub RH, Kalden JR. **Stress of different types increases the proinflammatory load in rheumatoid arthritis.** Arthritis Res Ther 2009;11:114-115.
19. Van Maanen MA, Vervoordeldonk MJ, Tak PP. **The cholinergic ant-inflammatory pathway: towards innovative treatment of rheumatoid arthritis.** Nat Rev Rheumatol 2009;229-232.
20. de Brouwer, S.J., Kraaimaat, F.W., Sweep, F.C. et al. **Experimental stress in inflammatory rheumatic diseases: a review of psychophysiological stress responses**. Arthritis Res Ther 12, R89 2010. https://doi.org/10.1186/ar3016
21. Thayer JF, Sternberg EM. **Neural aspects of immunomodulation: focus on the vagus nerve.** Brain Behav Immun 2010;24(8):1223-1228.
22. Waldburger JM, Firestein GS. **Regulation of peripheral inflammation by the central nervous system.** Curr Rheumatol Rep 2010;12:370-378.
23. Bruchfeld A, Goldstein RS, Chavan S, Patel NB, Rosas-Ballina M, Kohn N, Qureshi AR, Tracey KJ. **Whole blood cytokine attenuation by cholinergic agonists *ex vivo* and relationship to vagus nerve activity in rheumatoid arthritis.** J Intern Med 2010;268(1):94-101.
24. Huston JM, Tracey KH. **The pulse of inflammation: heart rate variability, the cholinergic anti-inflammatory pathway and implications for therapy.** J Intern Med 2011;269:45-53.
25. Koopman FA, Stoof SP, Straub RH, van Maanen MA, Vervoordeldonk MJ, Tak PP. **Restoring the balance of the autonomic nervous system as an innovative approach to the treatment of rheumatoid arthritis.** Mol Med 2011;17(9-10):937-948.
26. Dustin M. **Signaling at neuro/immune synapses.** J Clin Invest 2012;122:1149-1155.
27. Dias da Silva, VJ, Paton JFR. **The interplay between the autonomic and immune systems.** Exp Physiol 2012:97:1143-45.
28. Yadav RK, Gupta R, Deepak KK. **A pilot study on short term heart rate variability & its correlation with disease activity in Indian patients with rheumatoid arthritis.** Indian J Med Res. 2012 Oct;136(4):593-8. PMID: 23168699; PMCID: PMC3516026.
29. Ebbinghaus M, Gajda M, Boettger MK, Schaible HG, Brauer R. **The anti-inflammatory effects of sympathectomy in murine antigen-induced arthritis are associated with reduction of Th1 and Th17 responses.** Ann Rheum Dis 2012;71:253-261.
30. Straub RH**. Stress in RA: a trigger of proinflammatory pathways?** Nat Rev Rheumatol 2014:10(9):516-8.
31. Adlan AM, Lip GYH, Paton JFR, Kitas GD, Fisher JP. **Autonomic function and rheumatoid arthritis – a systematic review.** Semin Arthritis Rheum 2014; Dec;44(3):283-304.
32. Koopman FA, Tang MW, Vermeij J, de Hair MJ, Choi IY, Vervoordeldonk MJ, Gerlag DM, Karemaker JM, Tak PP. **Autonomic Dysfunction Precedes Development of Rheumatoid Arthritis: A Prospective Cohort Study.** EBioMedicine. 2016 Apr;6:231-237. doi: 10.1016/j.ebiom.2016.02.029. Epub 2016 Feb 19. PMID: 27211565; PMCID: PMC4856742.
33. Koopman FA, Chavan SS, Miljko S, Grazio S, Sokolovic S, Schuurman PR, Mehta AD, Levine YA, Faltys M, Zitnik R, Tracey KJ, Tak PP. **Vagus nerve stimulation inhibits cytokine production and attenuates disease severity in rheumatoid arthritis.** PNAS 2016;113(29): 8284-9.
34. Adlan AM, Veldhuijzen van Znaten JJCS, Lip GYH, Paton JFR, Kitas JD, Fisher JP. **Cardiovascular autonomic regulation, inflammation and pain in rheumatoid arthritis.** Autonomic Neuroscience: Basic and Clinical 2017;208:137–145.
35. Koopman FA, van Maanen MA, Vervoordeldonk MJ, Tak PP. **Balancing the autonomic nervous system to reduce inflammation in rheumatoid arthritis.** J Intern Med. 2017 Jul;282(1):64-75. doi: 10.1111/joim.12626. Epub 2017 May 26.
36. Rasmussen SE, Pfeiffer-Jensen M, Drewes AM, Farmer AD, Deleuran BW, Stengaard-Pedersen K, Brock B, Brock C. **Vagal influences in rheumatoid arthritis.** Scand J Rheum 2018;47(1):1-11.
37. Zimmermann M, Vodicka E, Holman AJ, Garrison LP**. Heart rate variability testing: could it change spending for rheumatoid arthritis patients in the United States? An exploratory economic analysis.** J Med Econ. 2018 Jul;21(7):712-720.
38. Taylor PC, Holman AJ. **Rheumatoid arthritis and the emergence of immuno-autonomics.** Rheumatology (Oxford). 2019 Dec 1;58(12):2079-2080. doi: 10.1093/rheumatology/kez216. PMID: 31177267.
39. Lin CY, Tsai SJ, Peng CK, Yang AC. **Sleep state instabilities in patients with periodic limb movements in sleep - Detection and quantification with heart rate variability**. Psychiatry Research, 2020 v293. doi:10.1016/j.psychres.2020.113454.
40. Ingegnoli F, Buoli M, Antonucci F, Coletto LA, Esposito CM and Caporali R. **The Link Between Autonomic Nervous System and Rheumatoid Arthritis: From Bench to Bedside.** Front. Med. 2020;7:589079. doi: 10.3389/fmed.2020.589079.
41. Martinez-Lavin M, Holman AJ. **Heart Rate Variability Analysis in Rheumatology: Past, Present…and Future?** [editorial] Clin Exp Rheum 2021 (in press).
42. Based on this information, how much do you agree or disagree with each of the following statements about the autonomic nervous system (ANS) and rheumatoid arthritis? **[ROTATE]**

|  | **Completely disagree** | **Somewhat disagree** | **Neither agree nor disagree** | **Somewhat agree** | **Completely agree** |
| --- | --- | --- | --- | --- | --- |
| ANS state may interfere with disease control in RA patients. | | | | | |
| Autoimmune diseases such as RA may be influenced by ANS state. | | | | | |
| Knowing ANS state could be helpful to rheumatologists to better treat their patients. | | | | | |
| I would like to be able to easily and accurately measure ANS state in RA patients. | | | | | |

**For the next set of questions, please consider the following information about heart rate variability (HRV) as an indicator of autonomic nervous system (ANS) state, and its applications to rheumatoid arthritis:**

Heart rate variability (HRV) is a well-established metric to assess ANS state. Cardiac rhythm is not static, even at rest (Shaffer et. al, 2014). Controlled by the ANS and mediated through aortic baroreceptors, heart rate fluctuates with breathing (respiratory sinus arrhythmia), leading to a continuous variability in the heart rate. Inhalation increases heart rate, while exhalation decreases heart rate (van der Klok, 2014). The degree of HRV reflects “the relative balance between the sympathetic and the parasympathetic systems” and can be used as a way to gauge the overall state of the autonomic nervous system (van der Klok, 2014).

Patients with lower HRV have a more active sympathetic system, while patients with higher HRV have a more active parasympathetic system.

- **Five-minute HRV is an internationally established metric of ANS state and can be measured quantitatively.** HRV was standardized as a measure of ANS state in 1996 (Task Force of the European Society of Cardiology and the North American Society of Pacing and Electrophysiology). Since then, the development of next-gen, high-fidelity instruments has made HRV measurements extremely precise (next-gen HRV is captured at 8,000 hertz with precision to 0.001 seconds). Next-gen HRV instruments measure both parasympathetic and sympathetic activity, providing a quantitative measure of these parameters for the first time.
-
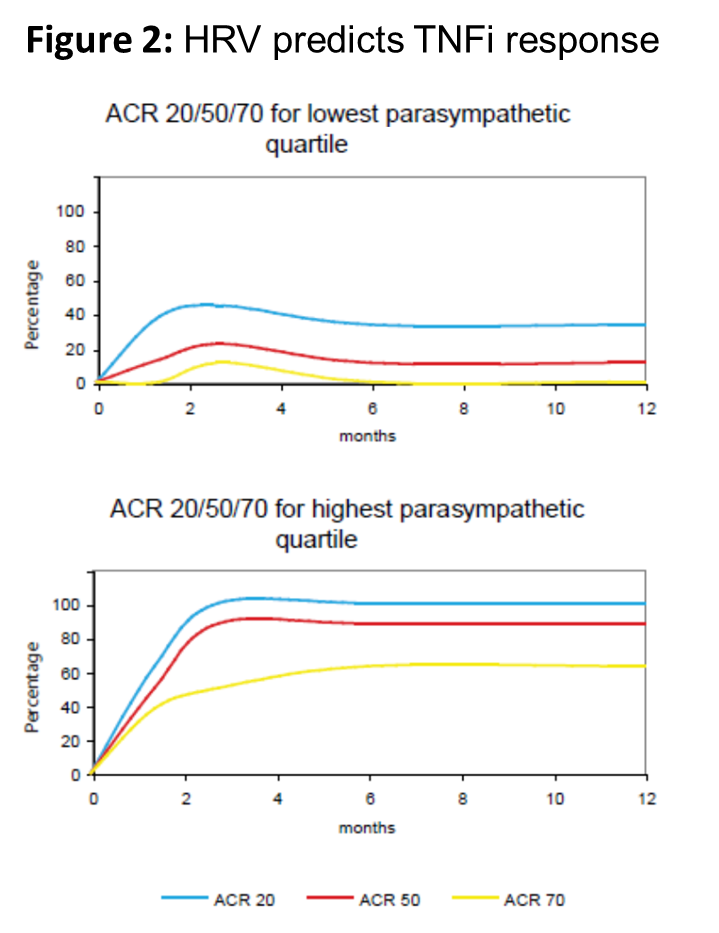
**HRV values may precede RA onset and correlate with established RA disease activity.** Koopman et al. (2016) found that autonomic dysfunction, as measured by HRV, may “precede and predict the development of rheumatoid arthritis.” In Koopman’s study, RA patients were shown to have lower parasympathetic activity, as measured by HRV. Yadev et al. (2012) reported a similar finding: “HRV was significantly altered in patients with RA and independently associated with disease activity.” Anichkov et al. (2007) also found that “reduced HRV is independently associated with high disease activity” among RA patients.
- **HRV predicts response to TNF inhibitors (TNFi) in RA patients.** Holman and Ng (2008) reported that next-gen HRV predicted 52-week rheumatoid arthritis TNFi ACR 20/50/70 outcomes with 90% sensitivity and 95.7% specificity in a prospective, double-blind study. See Figure 2 for an illustration of the findings. The top graph shows ACR responses among patients with unfavorable autonomic profile (lowest parasympathetic quartile). Fewer patients achieved ACR 20/50/70 responses compared to patients with favorable ANS profile (highest parasympathetic quartile, bottom graph).
- **
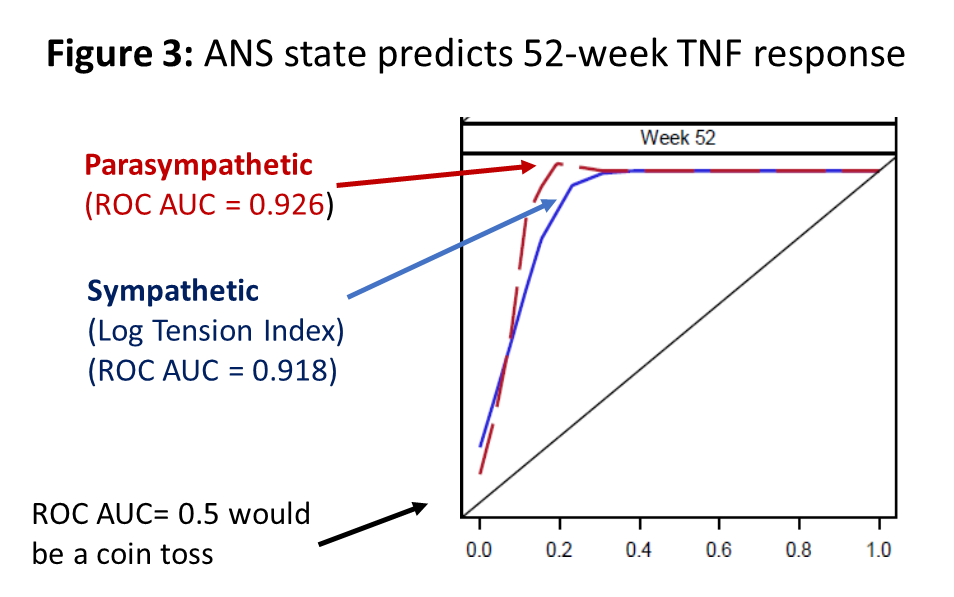
ANS state as measured via HRV was predictive of TNF response at 52 weeks.** ROC AUC curves were used to predict ACR 70 response to TNF using next-gen, high-fidelity HRV in a double-blind, prospective trial (Holman and Ng, 2015). Lower parasympathetic and higher sympathetic function ROC AUC were 0.926 and 0.918, respectively. See Figure 3 for more information. ROC curves farther to the left are indicative of a better association between autonomic state and response to therapy.
- **Treatments that improve ANS state improve outcomes for RA patients.** Vagal nervous stimulation inhibits cytokine production and attenuates disease severity in rheumatoid arthritis patients (Koopman et al., 2016). Marsal et al. (2021) likewise found that a noninvasive vagus nerve stimulation produced a clinically meaningful reduction in RA symptoms. Treatment of sleep apnea can also improve RA symptoms (Shimizu et al., 2003). Use of medications for restless leg syndrome can improve autonomic performance (Lin et al., 2020).
- **Several companies are developing treatments targeting the autonomic nervous system for application in autoimmune diseases.** Companies include SetPoint Medical and Galvani with implantable vagus nerve stimulation (VNS), Nesos with external/non-invasive VNS, and GSK and Inmedix with small molecules.

The overall model, showing the connection between reduced HRV and suboptimal therapeutic immunosuppression, is below:


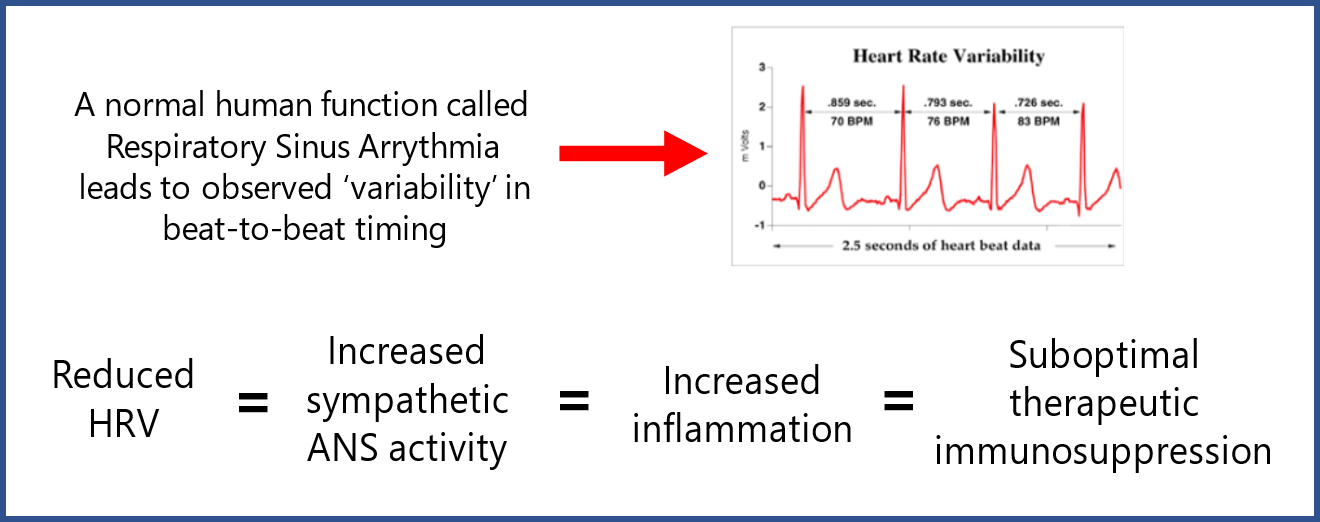


1. Assume there was an accurate, reliable five-minute test to measure the state of an RA patient’s autonomic nervous system (stress biology). You could use this test in-office and get immediate results, and the test would be reimbursable by insurance to the rheumatologist. How interested would you be in this test?

() Extremely interested

() Very interested

() Moderately interested

() Slightly interested

() Not at all interested

1. Based on the information provided, how much do you agree or disagree with each of the following statements about the autonomic nervous system and HRV? **[ROTATE]**

|  | **Completely disagree** | **Somewhat disagree** | **Neither agree nor disagree** | **Somewhat agree** | **Completely agree** |
| --- | --- | --- | --- | --- | --- |
| I would like to be able to quantitatively measure autonomic performance, including sympathetic and parasympathetic activity. | | | | | |
| Being able to quantitatively measure autonomic state (stress biology) could be useful to me as a rheumatologist in the care of my patients. | | | | | |
| Patients with poor autonomic function may be at risk for not responding adequately to conventional, biologic, or targeted synthetic DMARDs. | | | | | |
| I am interested in identifying RA patients with poor autonomic function (reduced parasympathetic activity and elevated sympathetic activity). | | | | | |

**[DIAGNOSTIC DEVICE]**

Thank you. Now we want to share a description and an image of a new device that measures the state of the autonomic nervous system using next-gen, high-fidelity heart rate variability (HRV) technology.


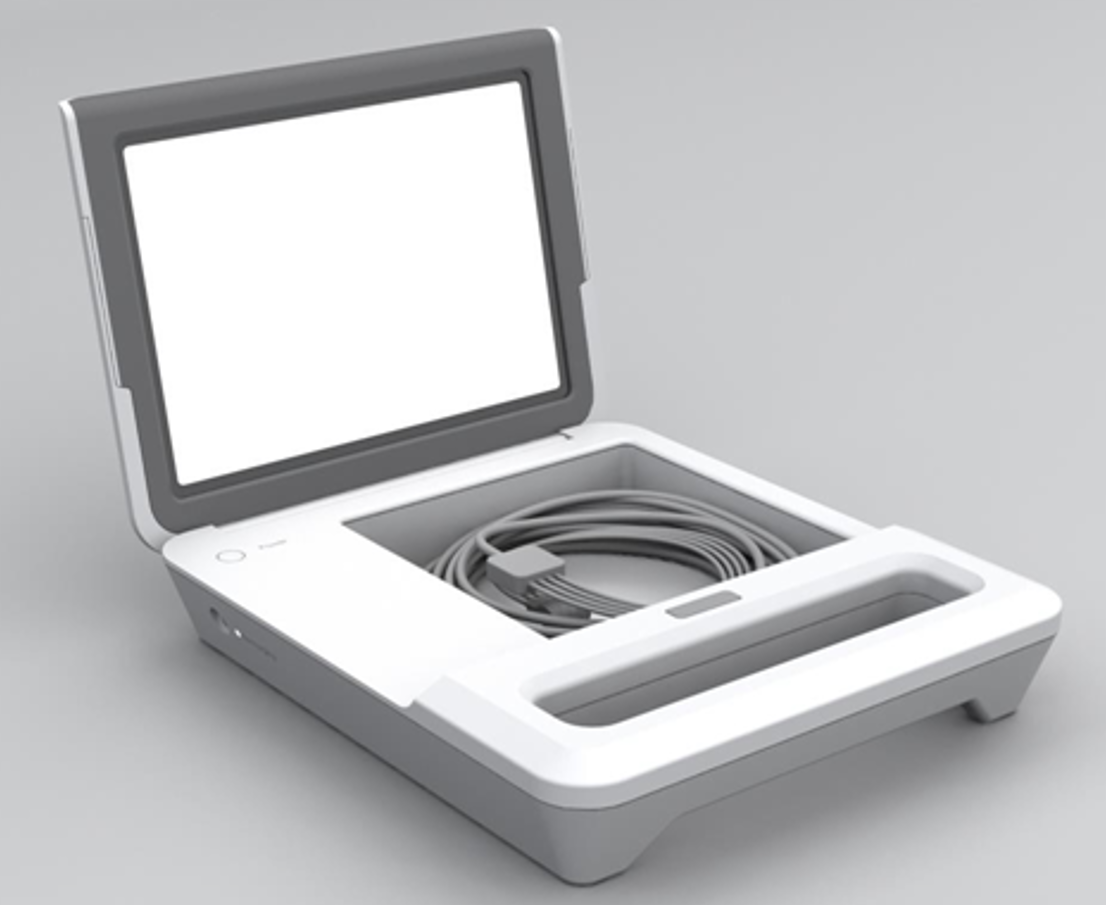
The device allows clinicians to accurately and easily measure the performance of the autonomic nervous system using next-generation HRV. This allows for a fast and accurate assessment of stress biology that can be reliably captured in a clinical setting.

The device is the size of a laptop. It consists of a processing unit and a removable iPad, which serves as a screen (see photo). To administer the test, the patient is asked to lie in a supine position, and four disposable ECG pads are placed on the patient’s wrists and ankles (patient clothing and shoes remain on). The device collects a five-minute ECG rhythm strip. Data are sent to the cloud for immediate processing and reporting.

Results include a quantitative assessment of autonomic nervous system performance (including numeric measurements of parasympathetic and sympathetic function) and are available immediately. The test is covered by current diagnostic codes and is reimbursable by third-party payers (e.g., Medicare reimburses at $91 to the rheumatologist).

1. Based on this information, how much do you agree or disagree with each of the following statements about the device? **[ROTATE]**

|  | **Completely disagree** | **Somewhat disagree** | **Neither agree nor disagree** | **Somewhat agree** | **Completely agree** |
| --- | --- | --- | --- | --- | --- |
| The device seems easy to use. | | | | | |
| I want to learn more about the device. | | | | | |
| The device may be helpful for the management of RA patients. | | | | | |
| The device quantitatively measures the state of the autonomic nervous system. | | | | | |

1. How interested would you be in using this device with your rheumatoid arthritis patients?

() Extremely interested

() Very interested

() Moderately interested

() Slightly interested

() Not at all interested

1. Which of the following factors make you interested in using the device with your RA patients? Please select all that apply. **[ROTATE ALL EXCEPT ‘OTHER’ AND ‘NONE’]**

[] Results are available immediately, in-office

[] Device provides a new source of revenue (Medicare pays $91 to rheumatologist)

[] Device measures ANS state

[] Device measures stress biology

[] Device is easy to use

[] Device will help my patients

[] Device may allow me to provide personalized medicine

[] Other: Please specify ___________

[] None of the above

1. For which group(s) of rheumatoid arthritis patients would you consider using the device? Please select all that apply.

[] All patients **[IF SELECTED, SELECT ALL BUT ‘NONE’ BELOW]**

[] Newly diagnosed patients at the beginning of their disease

[] Patients with loss of response to conventional, biologic, or targeted synthetic DMARDs

[] Patients for whom I am considering advancing therapy

[] Patients for whom I am considering tapering therapy

[] Patients for whom it is difficult to reach remission or low disease activity

[] Other: Please specify ___________

[] None of the above

1. For what percentage of your rheumatoid arthritis patients would you consider using the device at some point in their care?

() 0% **[IF SELECTED, SKIP QUESTION 21 AND GO TO DEMOGRAPHIC QUESTIONS]**

() 1% to 19%

() 20% to 39%

() 40% to 59%

() 60% to 79%

() 80% to 99%

() All (100%)

1. For patients for whom you would consider using the device, how often would you use it?

() Four or more times a year

() Three times a year

() Twice a year

() Annually

() Less often than annually

**[DEMOGRAPHIC QUESTIONS]**

Thank you for your responses. The following questions are for demographic purposes only.

1. What is your gender?

() Male

() Female

() Other

() Prefer not to answer

1. What is your race or ethnicity? Please select all that apply.

[] White

[] Black or African American

[] Hispanic or Latino

[] American Indian or Alaska Native

[] Asian

[] Native Hawaiian or Other Pacific Islander

[] Other

[] Prefer not to answer

1. What is your practice type?

() Solo

() Single specialty

() Multispecialty

1. Which of the following apply to your practice? Please select all that apply.

[] My practice is connected with a hospital/hospital system

[] My practice is part of an IDN (integrated delivery network)

[] My practice is part of an ACO (accountable care organization)

[] My practice is part of a GPO (group practice organization)

[] None of the above

1. What is your practice’s geographic location?

() Rural (fewer than 50,000 people)

() Suburban (50,000 to 149,999 people)

() Urban (150,000 or more people)

1. Which region of the country do you practice in?

() Midwest (IA, IL, IN, KS, MI, MN, MO, ND, NE, OH, SD, WI)

() Northeast (CT, DC, DE, MA, MD, ME, NH, NJ, NY, PA, RI, VT)

() Southeast (AL, AR, FL, GA, KY, LA, MS, NC, SC, TN, VA, WV)

() Southwest (AZ, NM, OK, TX)

() West (AK, CA, CO, HI, ID, MT, NV, OR, UT, WA, WY)

1. Do you consider yourself an early adopter of medical advances, meaning you adopt new diagnostic tests, clinical guidelines, and medications before most of your peers?

() No

() Yes

1. How frequently do you attend rheumatology meetings or conferences?

() Multiple times per year

() Annually

() Every few years

() Rarely or never

1. How often do you participate in continuing medical education opportunities to learn about new advances in rheumatology?

() Multiple times per year

() Annually

() Every few years

() Rarely or never

1. Do you subscribe to any medical or rheumatology journals?

() Yes

() No

Thank you. We appreciate your time and assistance.
